# American Journal of Dental Science

**Published:** 1853-10

**Authors:** 


### AMERICAN JOURNAL OF DENTAL SCIENCE.

This valuable journal has recently obtained the services of A. S. Piggott, M. D., as Assistant Editor, more particularly as it regards the department of Chemistry and Metallurgy. These are very important branches, and we are glad that one so well qualified has assumed the duty of bringing these subjects intelligently and prominently before the profession. Several recent numbers of the Journal contain articles from his pen which bear marks of much investigation and research, and are well worth the careful study of the Dental practitioner. We welcome Dr. Piggott to our editorial fraternity

